# Acupuncture and Sleep Quality Among Patients With Parkinson Disease: A Randomized Clinical Trial

**DOI:** 10.1001/jamanetworkopen.2024.17862

**Published:** 2024-06-26

**Authors:** Mingyue Yan, Jingqi Fan, Xin Liu, Yingjia Li, Yuting Wang, Weiqiang Tan, Yuanyuan Chen, Jun He, Lixing Zhuang

**Affiliations:** 1 The First Clinical College of Guangzhou University of Chinese Medicine, Guangzhou, China; 2 Guangzhou University of Chinese Medicine, Guangzhou, China; 3 Panyu Hospital of Chinese Medicine, Guangzhou, China; 4 The First Affiliated Hospital of Guangzhou University of Chinese Medicine, Guangzhou, China

## Abstract

**Importance:**

Poor sleep quality greatly impairs quality of life and accelerates deterioration in patients with Parkinson disease (PD), but current remedies remain limited. Acupuncture, used as an adjunctive therapy with anti-Parkinson medications, has shown positive effects in patients with PD. However, high-quality clinical evidence to support the effectiveness of acupuncture for patients with PD and poor sleep quality is lacking.

**Objective:**

To assess the safety and efficacy of real acupuncture (RA) vs sham acupuncture (SA) as an adjunctive therapy for patients with PD who have poor sleep quality.

**Design, Setting, and Participants:**

This single-center randomized clinical trial was performed at The First Affiliated Hospital of Guangzhou University of Chinese Medicine in China from February 18, 2022, to February 18, 2023. Patients with PD and sleep complaints were recruited and randomized (1:1) to receive RA or SA treatment for 4 weeks. Data analysis was performed from April 12 to August 17, 2023.

**Intervention:**

Treatment with RA or SA for 4 weeks.

**Main Outcomes and Measures:**

The main outcome was the change in Parkinson Disease Sleep Scale (PDSS) scores measured at baseline, after 4 weeks of treatment, and at 8 weeks of follow-up.

**Results:**

Of the 83 participants enrolled, 78 (94.0%) completed the intervention and were included in the analysis. Their mean (SD) age was 64.1 (7.9) years; 41 (52.6%) were men and 37 (47.4%) were women. A significant increase in PDSS scores from baseline was observed for both the RA group (29.65 [95% CI, 24.65-34.65]; *P* < .001) and the SA group (10.47 [95% CI, 5.35-15.60]; *P* < .001). Compared with the SA group, the RA group had a significant increase in PDSS scores after 4 weeks of treatment (19.75 [95% CI, 11.02-28.49]; *P* < .001) and at 8 weeks of follow-up (20.24 [95% CI, 11.51-28.98]; *P* < .001).

**Conclusions and Relevance:**

In this randomized clinical trial, acupuncture proved beneficial in improving sleep quality and quality of life among patients with PD. These findings suggest that the therapeutic effects of acupuncture could continue for up to 4 weeks.

**Trial Registration:**

Chinese Clinical Trial Registry Identifier: ChiCTR2200060655

## Key Points

**Question:**

What is the efficacy of acupuncture in treating patients with Parkinson disease (PD) who have poor sleep quality?

**Findings:**

In this randomized clinical trial of 78 patients, those randomly assigned to receive real acupuncture (RA) vs sham acupuncture (SA) for 4 weeks had a statistically significant improvement in Parkinson Disease Sleep Scale scores compared with baseline. This improvement persisted for 4 weeks posttreatment in the RA group but not in the SA group.

**Meaning:**

These findings suggest that acupuncture combined with the standard of care may improve sleep quality among patients with PD.

## Teaser

This randomized clinical trial examines the safety and efficacy of acupuncture as an adjunctive therapy for patients with Parkinson disease with poor sleep quality.

## Introduction

Parkinson disease (PD), which is characterized by bradykinesia, muscular rigidity, rest tremor (frequency, 4-6 Hz), and postural instability, is one of the most common and complex neurological disorders.^1^ As PD advances, nonmotor symptoms may predominate and become associated with increased disability and reduced health-related quality of life (HRQOL).^2,3,4^ Sleep disorder, as part of the disease course and side effects of anti-Parkinson medication, is a prevalent nonmotor symptom in PD and is associated with a more severe PD clinical phenotype.^5,6,7,8^ Studies on PD have consistently shown that poor sleep is linked to accelerated deterioration in gait and dyskinesia^9,10,11^ and faster progress in mood and cognitive symptoms.^12^ Consequently, sleep quality significantly affects HRQOL and symptom burden in patients with PD.^13,14,15^

Current management options for sleep disorder among patients with PD remain limited. The primary approach is to optimize dopaminergic therapy, particularly if the sleep disorder is related to nocturnal motor symptoms.^16^ Medications such as benzodiazepines, sedative antidepressants, and antipsychotics are beneficial but frequently accompanied by side effects, including excessive daytime sleepiness, morning sedation, imbalance, or confusion.^17^ Evidence on safety and efficacy is lacking for nondrug treatments such as cognitive behavior therapy, acupuncture, light treatment, repetitive transcranial magnetic stimulation, and exercise.^16^ Further studies should focus on novel approaches to treat PD, with the goal of applying safe and effective therapies to alleviate sleep symptoms, decrease motor symptoms, and improve quality of life.

Acupuncture is a therapeutic technique involving the insertion of needles into acupoints to induce a *de qi* sensation and trigger therapeutic effects, and it has been used in China for thousands of years. When used as an adjunctive therapy with anti-Parkinson medications, acupuncture has shown positive effects in improving sleep quality and reducing motor symptoms in patients with PD.^18,19,20^ However, there is still insufficient high-quality clinical evidence to support its effectiveness due to small sample sizes, unclear reporting, and potential bias.^21^ Therefore, we conducted a double-blind randomized clinical trial to evaluate the efficacy and safety of acupuncture for the treatment of poor sleep quality among patients with PD. This trial compared the effectiveness of real acupuncture (RA) vs sham acupuncture (SA), and it also comprehensively evaluated improvements in sleep quality, anxiety, nonmotor symptoms, motor symptoms, and HRQOL among patients with PD experiencing comorbid sleep and motor symptoms.

## Methods

### Study Design

This single-center, double-blind randomized clinical trial was approved by the Ethics Committee of The First Affiliated Hospital of Guangzhou University of Chinese Medicine in China and was conducted from February 18, 2022, to February 18, 2023. The trial protocol is presented in Supplement 1, and a trial procedure diagram is presented in eTable 1 in Supplement 2. All participants provided written informed consent during a screening visit. The study followed the Consolidated Standards of Reporting Trials (CONSORT) reporting guideline.

### Participants

Participants were recruited from the Parkinson Clinic of The First Affiliated Hospital of Guangzhou University of Chinese Medicine. Patients were included if they met the following criteria: (1) had a diagnosis of idiopathic PD according to the 2015 Movement Disorder Society clinical diagnostic criteria^22^; (2) self-reported moderate or severe sleep problems or a Parkinson Disease Sleep Scale (PDSS) score between 0 and 110; (3) were aged 30 to 80 years; (4) had Hoehn and Yahr stage 1 to 3; (5) accepted acupuncture therapy; (6) maintained stable use of anti-Parkinson medication over 30 days; and (7) understood the protocol and signed the informed consent form. Patients were excluded for the following reasons: they did not meet any of the inclusion criteria; were unable to cooperate due to severe cognitive dysfunction, blindness, or deafness; had PD comorbid with other serious systemic diseases such as stroke, malignant tumors, or kidney failure; used sleep-assisted medication irregularly; had received acupuncture within the last 30 days; had a history of drug or alcohol abuse; or were pregnant or lactating.

### Randomization and Blinding

Randomization was performed before baseline assessment. Eligible participants were randomly assigned to treatment with either RA or SA in a 1:1 ratio. An independent mathematician used SPSS Statistics, version 26.0 (IBM Corp), to produce a randomized sequence, and a third party concealed the allocation sequence in sealed opaque envelopes. Both participants and data analysts remained blinded to the treatment assignment throughout the study.

To achieve the double-blind acupuncture study, Wang et al^23^ designed an auxiliary acupuncture device to address challenges posed by current SA devices. This innovative device not only enables adjustment of the angle and direction of acupuncture, it also simulates the blocking sensation during needle entry. It fits tightly to the skin and can be applied to most parts of the human body, ultimately realizing the goal of blinding patients. The auxiliary acupuncture device is detailed in eFigures 1 and 2 and eTable 2 in Supplement 2.

### Acupuncture Procedures

All participants received sleep hygiene guidance from sleep clinic physicians and maintained their initial dosage of anti-Parkinson medication throughout the study as their standard treatment. An observer (Y.L.) meticulously recorded the type of anti-Parkinson medication, calculated the levodopa equivalent dose, and noted any drug changes required for participants adjusting their dosage.

Patients in the experimental group received RA, whereas those in the control group received SA. The same acupoint locations, including bilateral connections, were selected for both groups as follows: Si Shenzhen,^24^ ShenTing (GV24), YinTang (GV29), HeGu (LI4), TaiChong (LR3), SanYinJiao (SP6), ShenMen (HT7), ZuSanLi (ST36), ShenMai (BL62), and ZhaoHai (KI6) (eFigure 3 in Supplement 2). Acupoint names and locations adhered to the *National Standard of the People’s Republic of China Nomenclature and Location of Meridian Points (GB/T 12346-2021)*, established in 2021.^25^ Real acupuncture was administered with single-use, sterilized, stainless steel needles (0.25 × 25 mm, 0.25 × 40 mm; Tianxie). Sham acupuncture was administered using specially designed sham stainless steel needles lacking a sharp tip, rendering it difficult to pierce the skin and enter the subcutaneous tissue.

Acupuncture was administered 3 times per week (every Monday, Wednesday, and Friday) for 4 weeks. During each 30-minute session, every patient assumed a supine position and wore an opaque eye mask. After acupoint positioning and routine disinfection, physicians pressed the needle holder tightly to the skin of the acupoints and then performed acupuncture, inserting real or sham needles into the corresponding 15°/90° needle entry portals on the needle holders swiftly and painlessly. Finally, the needle holes were pressed with a sterilized dry cotton swab briefly after needle removal.

### Clinical Assessments

#### Primary Outcome

The primary outcome was the change in PDSS scores assessed at 3 time points: at baseline, after 4 weeks of treatment, and at 8 weeks of follow-up. The PDSS is a 15-item scale that gauges self-reported effects of poor sleep quality on various functions, evaluating 8 aspects of nocturnal sleep in PD.^26^ These aspects include the overall quality of a night’s sleep, insomnia, nocturnal restlessness, nocturnal psychosis, nocturia, nocturnal motor symptoms, sleep refreshment, and daytime dozing. The assessed period is the past 1 week, with patients marking their response to each item on a visual analog scale ranging from 0 (always) to 10 (never). Total possible PDSS scores range from 0 to 150, and scores increase as sleep quality improves.

#### Secondary Outcomes

Secondary outcomes included the treatment completion rate, adverse events (AEs), and participant outcome assessment. The assessment comprised the following protocol: First, motor symptom severity was quantified with the modified Hoehn and Yahr scale, the Unified Parkinson Disease Rating Scale (UPDRS and UPDRS section III [UPDRS-III]), and the daily levodopa equivalent dose. Second, overall severity of nonmotor symptoms was assessed with the Non-Motor Symptoms Scale (NMSS). Specifically, excessive daytime sleepiness was assessed with the Epworth Sleepiness Scale (ESS) and anxiety levels were assessed with the Hamilton Anxiety Rating Scale (HAM-A). Finally, quality of life was assessed with the 39-item Parkinson Disease Questionnaire (PDQ-39). Key secondary outcomes were also assessed at 3 time points: at baseline, at 4 weeks posttreatment, and at 8 weeks of follow-up. The primary and secondary outcomes are presented in eTable 3 in Supplement 2.

We also monitored and recorded treatment-induced AEs, such as needle sickness, needle breakage, hematoma, and infection. A severe AE was defined as any AE posing a threat to a patient’s life or functioning. The study investigators assessed AE severity (mild, moderate, or severe). Acupuncturists should evaluate the patient’s condition to decide whether the treatment can be continued if AEs occur. This study describes the number and proportion of AEs observed.

### Statistical Analysis

The sample size was based on our pilot study with mean (SD) PDSS scores of 122.3 (15.4) for the RA group and 100.4 (21.4) for the SA group. To achieve 90% power at a 2-sided significance level of *P* < .05, a sample size of 44 patients (22 per group) was calculated. Considering a 20% dropout rate, the sample size was 56 patients as calculated with PASS software, version 15.0.5 (NCSS).

Descriptive analysis was used for baseline characteristics of patients in each group. For continuous variables, Shapiro-Wilk normality analysis was applied at baseline, with results conveyed as the mean (SD); the *t* test was used for normal distributions, with median values presented; and the Mann-Whitney *U* test was used for nonnormal distributions (Table 1). The χ^2^ test was used for categorical variables. Outcome scales were assessed with a linear mixed-effects model with the PROC MIXED data procedure in SAS, version 9.4 (SAS Institute Inc), to investigate the effect of treatment group, time, and their interaction on the outcome measures, while also considering random effects among patients. Multiple imputations were used for missing data with the PROC MI data procedure in SAS. Efficacy was assessed in the full analysis set, which included all randomized patients who received at least 1 week of acupuncture. Continuous variables are presented as least-squares means with 95% CIs. It is important to note that the 95% CIs were not adjusted for multiple comparisons and should not be used to infer definitive treatment effects.^27^ Two-sided *P* < .05 was considered statistically significant. Data analysis was performed from April 12 to August 17, 2023.

**Table 1.** Baseline Demographic and Clinical Characteristics of Patients^a^

Characteristic	Patients (N = 78)	RA group (n = 40)	SA group (n = 38)	*P* value
Age, mean (SD), y	64.1 (7.9)	64.4 (7.5)	63.8 (8.5)	.75
Sex				
Male	41 (52.6)	23 (57.5)	18 (47.4)	.37
Female	37 (47.4)	17 (42.5)	20 (52.6)	
Alcohol consumption history				
Yes	20 (25.6)	11 (27.5)	9 (23.7)	.52
No	58 (74.4)	29 (72.5)	29 (76.3)	
Smoking history				
Yes	20 (25.6)	9 (22.5)	11 (28.9)	.70
No	58 (74.4)	31 (77.5)	27 (71.1)	
PD severity by modified Hoehn and Yahr stage				
<2.5	54 (69.2)	29 (72.5)	25 (65.8)	
2.5	12 (15.4)	7 (17.5)	5 (13.2)	
3	12 (15.4)	4 (10.0)	8 (15.4)	
Levodopa equivalent dose, mean (SD), mg/d	613.6 (326.0)	621.3 (361.1)	605.6 (289.1)	.83
Duration of PD, mean (SD), y	7.2 (4.9)	7.9 (5.7)	6.5 (3.8)	.21
Score on outcome measure, mean (SD)				
PDSS	85.3 (19.0)	85.6 (3.1)	85.0 (3.0)	.90
Sleep quality rating				
0-60	42 (53.8)	22 (55.0)	20 (52.6)	NA
61-90	24 (30.8)	12 (31.6)	12 (31.6)	NA
91-110	12 (15.4)	6 (15.8)	6 (15.8)	NA
ESS	8.7 (5.7)	8.8 (0.9)	8.6 (1.0)	.85
UPDRS	44.3 (20.2)	44.2 (2.7)	44.4 (3.8)	.97
UPDRS-III	24.8 (11.7)	24.7 (1.7)	24.8 (2.1)	.97
NMSS	39.8 (18.6)	39.5 (2.2)	40.1 (3.7)	.87
HAM-A	14.3 (6.2)	13.9 (1.0)	14.6 (1.0)	.53
PDQ-39	38.6 (17.1)	39.2 (3.1)	38 (2.3)	.71

Abbreviations: ESS, Epworth Sleepiness Scale; HAM-A, Hamilton Anxiety Rating Scale; NA, not applicable; NMSS, Non-Motor Symptoms Scale; PD, Parkinson Disease; PDQ-39, 39-item Parkinson Disease Questionnaire; PDSS, Parkinson Disease Sleep Scale; RA, real acupuncture; SA, sham acupuncture; UPDRS, Unified Parkinson Disease Rating Scale; UPDRS-III, section 3 of the Unified Parkinson Disease Rating Scale.

^a^ Unless indicated otherwise, values are presented as No. (%) of patients.

## Results

**Figure 1.**
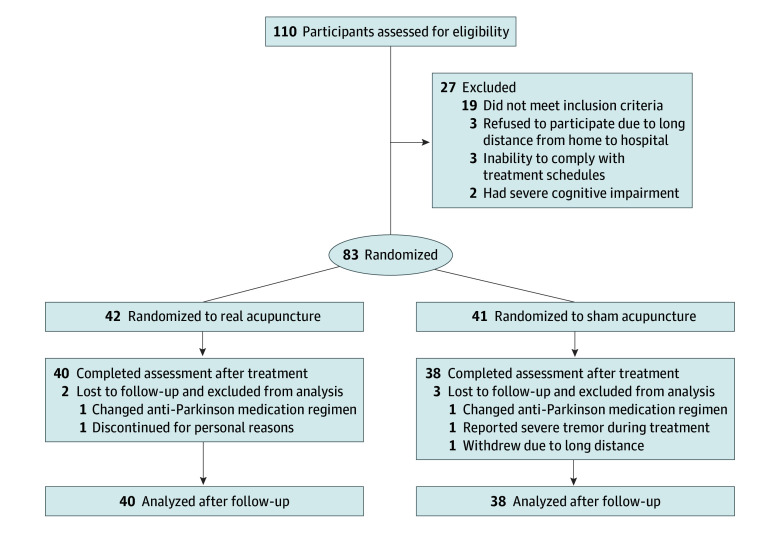
Study Flowchart

Of the 83 participants enrolled and randomly assigned to treatment, 78 (94.0%; 40 in the RA group and 38 in the SA group) successfully completed the intervention and the 4-week follow-up (Figure 1). Table 1 presents the baseline demographic and clinical characteristics of the 78 included participants. Their mean (SD) age was 64.1 (7.9) years; there were 41 men (52.6%) and 37 women (47.4%). Five patients (6.0%) dropped out due to adjustment of anti-Parkinson medication or for personal reasons. Multiple imputation was used for missing data for 7 participants (2 in the RA group and 5 in the SA group).

### Baseline Characteristics

#### Primary Outcome

**Figure 2.**
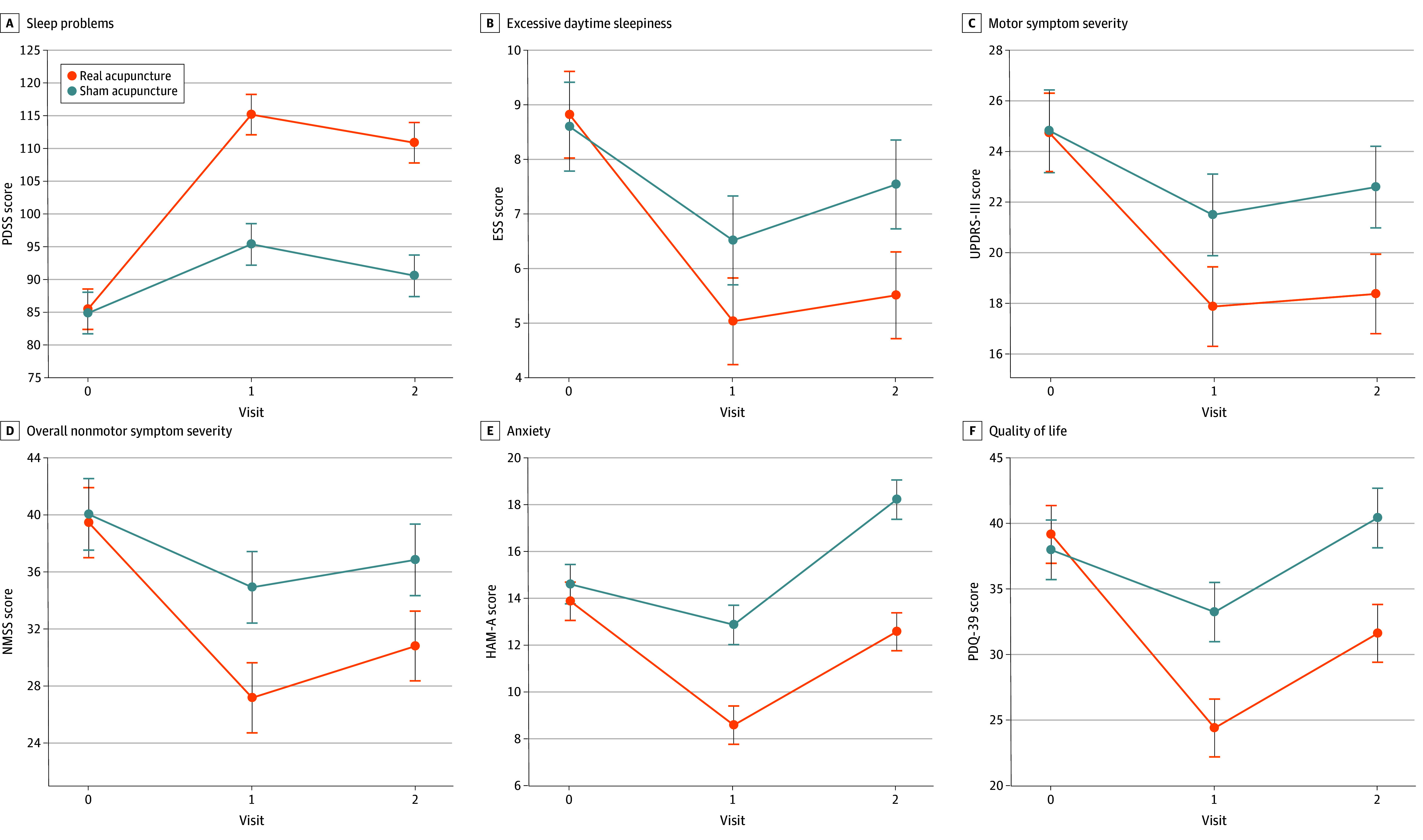
Therapeutic Effects of Acupuncture Over Time A, Sleep problems were evaluated using the Parkinson Disease Sleep Scale (PDSS). B, Excessive daytime sleepiness was assessed with the Epworth Sleepiness Scale (ESS). C, Motor symptom severity was evaluated with section 3 of the Unified Parkinson Disease Rating Scale (UPDRS-III). D, Overall nonmotor symptom severity was assessed with the Non-Motor Symptoms Scale (NMSS). E, Anxiety levels were assessed with the Hamilton Anxiety Rating Scale (HAM-A). F, Quality of life was assessed with the 39-item Parkinson Disease Questionnaire (PDQ-39). Scores are presented with 95% CIs.

The RA and SA groups demonstrated statistically significant improvement at 4 weeks posttreatment and at 8 weeks of follow-up. Mean PDSS scores increased significantly from baseline for the RA group (29.65 [95% CI, 24.65-34.65]; *P* < .001) and the SA group (10.47 [95% CI, 5.35-15.60]; *P* < .001; Table 2). Notably, patients in the RA group had a significantly greater increase in PDSS scores posttreatment (19.75 [95% CI, 11.02-28.49]; *P* < .001) and during follow-up (20.24 [95% CI, 11.51-28.98]; *P* < .001) compared with the SA group (Table 3). Figure 2 illustrates the changes in PDSS scores between the 2 groups.

**Table 2.** Treatment Effects of RA and SA From Baseline to 4 Weeks Posttreatment and 8 Weeks of Follow-Up

Outcome assessment	RA group (n = 40)		SA group (n = 38)	
	Change from baseline, mean (95% CI)	*P* value	Change from baseline, mean (95% CI)	*P* value
PDSS				
Posttreatment	29.65 (24.65 to 34.65)	<.001	10.47 (5.35 to 15.60)	<.001
Follow-up	25.35 (20.35 to 30.35)	<.001	5.68 (0.56 to 10.81)	.03
ESS				
Posttreatment	−3.78 (−4.82 to −2.73)	<.001	−2.08 (−3.15 to −1.01)	<.001
Follow-up	−3.30 (−4.34 to −2.26)	<.001	−1.05 (−2.12 to 0.02)	.05
UPDRS				
Posttreatment	−14.53 (−17.73 to −11.32)	<.001	−2.95 (−6.24 to 0.35)	.08
Follow-up	−14.28 (−17.48 to −11.07)	<.001	−0.58 (−3.87 to 2.71)	.73
UPDRS-III				
Posttreatment	−6.88 (−9.01 to −4.73)	<.001	−3.34 (−5.53 to −1.14)	.003
Follow-up	−6.38 (−8.51 to −4.23)	<.001	−2.24 (−4.43 to −0.04)	.046
NMSS				
Posttreatment	−12.28 (−16.00 to −8.55)	<.001	−5.10 (−8.92 to −1.29)	.009
Follow-up	−8.65 (−12.37 to −4.92)	<.001	−3.18 (−7.00 to 0.63)	.10
HAM-A				
Posttreatment	−5.28 (−6.76 to −3.78)	<.001	−1.74 (−3.26 to −0.21)	.03
Follow-up	−1.30 (−2.79 to 0.19)	.09	3.61 (2.08 to 5.13)	<.001
PDQ-39				
Posttreatment	−14.75 (−18.62 to −10.88)	<.001	−4.74 (−8.70 to −0.77)	.02
Follow-up	−7.55 (−11.42 to −3.68)	<.001	2.42 (−1.54 to 6.39)	.23

Abbreviations: ESS, Epworth Sleepiness Scale; HAM-A, Hamilton Anxiety Rating Scale; NMSS, Non-Motor Symptoms Scale; PDQ-39, 39-item Parkinson Disease Questionnaire; PDSS, Parkinson Disease Sleep Scale; RA, real acupuncture; SA, sham acupuncture; UPDRS, Unified Parkinson Disease Rating Scale; UPDRS-III, section 3 of the Unified Parkinson Disease Rating Scale.

**Table 3.** Treatment Effects of RA and SA at 4 Weeks Posttreatment and 8 Weeks of Follow-Up

Variable	RA group, mean (SD) (n = 40)	SA group, mean (SD) (n = 38)	Difference (95% CI)	*P* value
Primary outcome				
PDSS				
Posttreatment	115.20 (16.99)	95.45 (20.26)	19.75 (11.02 to 28.49)	<.001
Follow-up	110.90 (18.68)	90.66 (22.47)	20.24 (11.51 to 28.98)	<.001
Secondary outcome				
ESS				
Posttreatment	5.05 (3.73)	6.53 (5.02)	−1.48 (−3.73 to 0.77)	.20
Follow-up	5.53 (4.04)	7.55 (5.41)	−2.03 (−4.23 to 0.22)	.08
UPDRS				
Posttreatment	29.70 (12.07)	41.42 (22.95)	−11.72 (−19.90 to −3.55)	.005
Follow-up	29.95 (11.55)	43.79 (19.54)	−13.84 (−22.01 to −5.67)	.001
UPDRS-III				
Posttreatment	17.85 (9.05)	21.47 (9.88)	−3.62 (−8.10 to 0.84)	.11
Follow-up	18.35 (8.72)	22.58 (7.94)	−4.23 (−8.70 to 0.24)	.06
NMSS				
Posttreatment	27.20 (12.18)	34.95 (15.75)	−7.75 (−14.69 to −0.80)	.03
Follow-up	30.83 (10.02)	36.87 (15.75)	−6.04 (−12.98 to 0.90)	.09
HAM-A				
Posttreatment	8.63 (4.16)	12.89 (4.91)	−4.27 (−6.58 to −1.96)	<.001
Follow-up	12.60 (4.79)	18.24 (4.22)	−5.64 (−7.95 to −3.33)	<.001
PDQ-39				
Posttreatment	24.43 (13.01)	33.26 (10.06)	−8.84 (−15.08 to −2.60)	.005
Follow-up	31.63 (13.12)	40.42 (11.36)	−8.80 (−15.04 to −2.56)	.006

Abbreviations: ESS, Epworth Sleepiness Scale; HAM-A, Hamilton Anxiety Rating Scale; NMSS, Non-Motor Symptoms Scale; PDQ-39, 39-item Parkinson Disease Questionnaire; PDSS, Parkinson Disease Sleep Scale; RA, real acupuncture; SA, sham acupuncture; UPDRS, Unified Parkinson Disease Rating Scale; UPDRS-III, section 3 of the Unified Parkinson Disease Rating Scale.

#### Secondary Outcomes

Statistically significant within-group differences in UPDRS, HAM-A, and PDQ-39 scores were observed at 4 weeks posttreatment and at 8 weeks of follow-up compared with baseline. A significant decrease in NMSS scores was observed posttreatment, but there was no significant difference during follow-up. No significant differences in ESS and UPDRS-III scores were observed posttreatment or during follow-up compared with baseline (Figure 2 and Table 2).

Compared with baseline, the RA group in this study exhibited significant decreases in scores on the following 6 outcome measures after the 4-week intervention: ESS (−3.78 [95% CI, −4.82 to −2.73]), UPDRS (−14.53 [95% CI, −17.73 to −11.32]), UPDRS-III (−6.88 [95% CI, −9.01 to −4.73]), NMSS (−12.28 [95% CI, −16.00 to −8.55]), HAM-A (−5.28 [95% CI, −6.76 to −3.78]), and PDQ-39 (−14.75 [95% CI, −18.62 to −10.88]). Compared with the SA group, the RA group had significant decreases in scores posttreatment on the UPDRS (−11.72 [95% CI, −19.90 to −3.55]; *P* = .005), NMSS (−7.75 [95% CI, −14.69 to −0.80]; *P* = .03), HAM-A (−4.27 [95% CI, −6.58 to −1.96]; *P* < .001), and PDQ-39 (−8.84 [95% CI, −15.08 to −2.60]; *P* = .005). At the end of treatment, the variance in changes in ESS and UPDRS-III scores between the 2 groups was not statistically significant (Figure 2, Table 3, and eFigure 4 in Supplement 2).

### Maintenance of Benefits

At 8 weeks of follow-up, the RA group had significant decreases in scores compared with the SA group on the UPDRS (−13.84 [95% CI, −22.01 to −5.67]; *P* = .001), HAM-A (−5.64 [95% CI, −7.95 to −3.33]; *P* < .001), and PDQ-39 (−8.80 [95% CI, −15.04 to −2.56]; *P* = .006). The variance in changes in ESS, UPDRS-III, and NMSS scores between the 2 groups was not statistically significant during follow-up (Table 3). Among the secondary outcome measures, no statistically significant improvements in motor symptoms and daytime sleepiness were observed.

### Adverse Events

No severe AEs occurred in either group. The most common self-reported acupuncture-related AEs were severe tremor; bleeding, numbness, or infection; and sharp pain during treatment (eTable 4 in Supplement 2). Non–acupuncture-related AEs occurred infrequently. No patterns of differences between the 2 groups were observed. All AEs were managed, and no participants withdrew from the study because of an AE.

## Discussion

This trial explored the efficacy of acupuncture among patients with PD (Hoehn and Yahr stage ≤3) and sleep problems. Participants in the RA group experienced a significant 29.65-point improvement in PDSS scores 4 weeks posttreatment, which persisted to 8 weeks of follow-up. This finding suggests that RA is an effective treatment for patients with PD and sleep problems. Patients in the SA group also had a significant 10.47-point improvement in PDSS scores at week 4, but this effect did not persist during follow-up. We preliminarily conclude that although SA may induce a short-term placebo effect, acupuncture provides lasting clinical benefits in improving subjective sleep quality in patients with PD. In this study, the placebo effect of acupuncture diminished gradually, but its therapeutic benefits remained over a prolonged period.

In the RA group, scores on all scale items, which encompass motor symptoms (UPDRS, UPDRS-III), nonmotor symptoms (NMSS, ESS, HAM-A), and HRQOL (PDQ-39), improved significantly after the intervention. Previous studies^28,29^ have established significant correlations between the PDSS and the PDQ-39, HAM-A, and ESS and a lower but still significant correlation of the PDSS with the UPDRS-III. Considering the interaction between sleep conditions and motor symptoms, as well as the effects of poor sleep on quality of life, acupuncture seems to enhance overall function and quality of life in participants with PD by alleviating sleep disturbances. In this study, this therapeutic effect of RA persisted over 4 weeks (except for anxiety, assessed with HAM-A scores), indicating benefits of acupuncture at the 4-week mark. The SA group also had statistically significant decreases in ESS, UPDRS-III, NMSS, and PDQ-39 scores, although these improvements were less pronounced than those in the RA group. Furthermore, placebo benefits were sustained for less than 4 weeks.

Data on AEs were comparable for both groups. No severe AEs occurred during the experiment, and all moderate AEs were well managed. Severe tremor during treatment was reported by some participants (RA, 7 [17.5%] vs SA, 9 [23.7%]), potentially due to anxiety associated with acupuncture, leading to heightened tension and exacerbated tremor symptoms. Typically, patients experience increased tremor within 10 minutes of needle insertion, which gradually subsides as patients calm down. In summary, acupuncture appears to be safe and effective for patients with PD and poor sleep quality.

To date, there have been no large-scale randomized clinical trials focused on the effects of acupuncture in alleviating sleep problems among patients with PD. In 2002, a nonblinded pilot study^30^ first confirmed the safety and tolerability of acupuncture therapy in PD, demonstrating improvement in sleep and rest among 20 patients. In 2022, a meta-analysis^31^ highlighted the benefits of acupuncture combined with medication for PD-related insomnia compared with medication alone or SA. Moreover, acupuncture was effective in improving cognition, quality of life, behavior, and mood. Since then, only 1 randomized clinical trial^32^ on acupuncture therapy has indicated the efficacy of acupuncture on sleep disturbance with NMSS and PDSS scores.

Acupuncture has been used in China for thousands of years. Previous studies^33,34^ have demonstrated its efficacy in treating insomnia, with symptom improvements lasting over 3 weeks. A functional magnetic resonance imaging study^35^ suggested that different brain mechanisms may be recruited in different acupuncture modalities. Acupuncture induces both specific and nonspecific effects, whereas SA produces only nonspecific effects.^36^ Compared with penetrating SA, noninvasive sham treatment may decrease the multisensory stimulations or nonspecific effects.^37,38^ Therefore, noninvasive SA would be excellent to reduce the nonspecific effects. To accomplish the goal of double-blind, noninvasive SA control, we created an auxiliary device that can conduct noninvasive acupuncture and adjust the needle insertion angle.^23^ This device, used in an environment closely resembling actual needling, minimizes bias and potential therapeutic effects, ensuring an accurate evaluation of needling effects. Successful blinding tests were conducted, with participants unaware of the treatment received.

### Strengths and Limitations

This study has several strengths. We used a robust double-blinded, randomized clinical trial design, enhancing the reliability and internal validity of the findings. We also used our auxiliary device to achieve blinding, which reduces the risk of performance and assessment bias, strengthening the overall quality of evidence. Furthermore, we incorporated a thorough set of outcome measures, including sleep metrics and crucial aspects affected by poor sleep, including motor function, nonmotor function, and overall quality of life, providing a holistic understanding of the effects of acupuncture on patients with PD and sleep disturbances.

This study also has several limitations. First, the nature of acupuncture resulted in the acupuncturists being unblinded. However, we minimized bias by assigning independent acupuncturists for RA and SA, with each participant wearing an opaque eye mask during therapy. Additionally, the follow-up period was limited to 4 weeks, potentially restricting the assessment of long-term effects and sustainability of acupuncture interventions. Longer-term follow-up could provide valuable insights on treatment durability and symptom relapse. Finally, participants were recruited from a specialty PD clinic in China, possibly limiting the generalizability of these findings. Future research should consider diverse participant samples to ensure the applicability of the results to the broader population of patients with PD.

## Conclusions

In this randomized clinical trial, acupuncture improved sleep quality and overall quality of life for individuals with PD. The therapeutic effects persisted for up to 4 weeks, underscoring the potential of acupuncture as a beneficial adjunct in managing sleep-related issues among patients with PD.

## Notes

Supplement 1.
Trial Protocol

Supplement 2. **eFigure 1.** Schematic Diagram of the Auxiliary Acupuncture Device **eFigure 2.** Auxiliary Acupuncture Device **eFigure 3.** Acupoint Locations **eFigure 4.** Therapeutic Effects of Acupuncture **eTable 1.** Procedure Diagram **eTable 2.** Blinding Assessment **eTable 3.** Primary and Secondary Outcomes **eTable 4.** Acupuncture-Related Adverse Events

Supplement 3.
Data Sharing Statement
