# Supplementary material for: Acupuncture and Sleep Quality Among Patients With Parkinson Disease: A Randomized Clinical Trial
Source: JAMA Netw Open. 2024 Jun 26;7(6):e2417862. doi: 10.1001/jamanetworkopen.2024.17862 (PMC11208974; doi:10.1001/jamanetworkopen.2024.17862)
Supplement: Supplement 1. — Trial Protocol [file jamanetwopen-e2417862-s001.pdf]

Acupuncture for Parkinson's disease patients with poor sleep quality: A randomized, controlled trial

The First Affiliated Hospital of Guangzhou University of Chinese Medicine,

Guangzhou University of Chinese Medicine

Data:

Original protocol date: Nov 08, 2021

Amendment date: Jan 18, 2022

Clinical sites:

The First Affiliated Hospital of Guangzhou University of Chinese Medicine

Data Management and Statistical Centers:

Guangzhou University of Chinese Medicine

Confidentiality Statement

This document is the intellectual property of the Investigators. The information provided in this document is strictly confidential and is available for review to the sponsor, investigators, potential investigators, appropriate Ethics Committees, Investigational Review Boards, and other government regulatory bodies. No disclosure should take place without written authorization from the protocol developing investigators, except to the extent necessarily needed to obtain informed consent from potential patients.

Table of Contents

1. Study Contact and Organization 4
1.1 Study Contacts 4
1.2 Investigators 4
2. Study Design 4
2.1 Study Overview 4
2.2 Background 5
2.3 Study Objective 5
2.4 Methodology 6
2.4.1 Trial Design 6
2.4.2 Patients 6
2.4.3 Trial Flow 7
2.4.4 Randomization and Allocation Concealment 8
2.4.5 Blinding 9
2.4.6 Sample Size 9
3. Interventions 9
3.1 Basic treatment 9
3.2 Real Acupuncture (RA groups) 10
3.3 Sham Acupuncture (SA group) 10
3.4 the Auxiliary Acupuncture Device 11
4. Outcome Measurements 11
4.1 Primary Outcomes 11
4.2 Secondary Outcomes 12
5. Safety Assessment 13
6. Ethical Principle 14
7. Statistical Analysis 14
8.Some revisions 14
9. Funding 15
10. References 15
Appendix 1 17

1. Study Contact and Organization

1.1 Study Contacts

Principal Investigator for study,
Professor Lixing Zhuang.
The First Affiliated Hospital of Guangzhou University of Chinese Medicine, No. 16, Airport Rd, Baiyun District, Guangzhou 510000, Guangdong, China.
Email: zhuanglixing@163.com

1.2 Investigators

Lixing Zhuang, Professor, supervisor of acupuncture scheme
Ming-yue Yan, PhD, Management and Implementation of experiment, Designer of acupuncture scheme
Jingqi Fan, PhD, Designer and supervisor of acupuncture scheme
Xin-Liu, PhD, Design and manufacture of sham needle, supervisor of acupuncture scheme
Yu-ting Wang, PhD, Design and manufacture of sham needle, Data analysis
Wei-qiang Tan, MM, Implementation of experiment
Weijing Lu，MM, Implementation of experiment
Yuan-yuan Chen, MM, Implementation of experiment
Qing-Liu, MM, Implementation of experiment
Xiaoyan Xu, Implementation of experiment
Ian-I Leong, MM, Outcome evaluation and data collection
Ying-jia Li, MM, Outcome evaluation and data collection
Jun-He, PhD, Professor, supervisor of acupuncture scheme

2. Study Design

2.1 Study Overview

This randomized controlled trial was conducted to assess the safety and efficacy of real acupuncture versus sham acupuncture as adjunctive therapy for PD patients with poor sleep quality. We hypothesized that real acupuncture(RA) combined with anti-parkinson medication would be an effective treatment for sleep and sleep-related symptoms of PD patients. This trial has been registered at Chinese Clinical Trial Registry (CHICTR2200060655).

2.2 Background

With the increase in the percentage of aged people in the global population, the incidence of Parkinson's disease (PD) is also increasing. Parkinson’s disease(PD) is characterized by four primary motor symptoms: Bradykinesia, Muscular rigidity,4–6 Hz rest tremor and Postural instability[1] . As the disease advances, non-motor symptoms (NMS) of Parkinson's disease can predominate and are associated with reduced health-related quality of life(HRQoL)[2-4]. PD patients, experiencing poor sleep quality and quantity, typically come to hospitals with sleep complaints and more severe motor conditions. Researches have shown that poor sleep in PD is linked to a more rapid deterioration in gait and dyskinesia[5-7].In other words, poor sleep is associated with a more severe PD clinical phenotype. Besides, mental state and movement medications can aggravate sleep concerns[8,9]. Additionally, subjective sleep quality can affect health-related quality of life in PD patients[10,11]. Therefore, paying much more attention to the management of sleep quality could be beneficial to addressing NMS, motor symptoms, and HRQoL in PD patients.

The health-related quality of life(HRQoL) in PD patients can be significantly affected by sleep disturbances. Current management options remain limited, primarily consisting of drug treatment, and non-drug treatment. After evaluating the cause and the subtype of sleep disorders(SDs), the first choice is to optimize dopaminergic therapy if the SD is related to nocturnal motor symptoms[12]. Drugs like benzodiazepines, sedative antidepressants and antipsychotics are beneficial while they are frequently accompanied by side effects such as morning sedation, imbalance, or confusion[13]. Non-drug treatments, such as cognitive-behavioral therapy, acupuncture, light treatment, repetitive transcranial magnetic stimulation, and exercise, provide insufficient evidence on their safety and efficacy[12]. Further studies should focus on novel approaches with the goal of applying safe and effective therapies to alleviate sleep symptoms, motor symptoms and improve quality of life in PD patients.

There is limited evidence of Non-drug treatments in the treatment of constipation in PD. Safe and effective complementary treatments for the motor and sleep symptoms of Parkinson's disease are required.

Acupuncture is a safe adjunctive therapy that has been used for the treatment of PD diseases and has shown positive effects in reducing motor symptoms and non-motor symptoms in PD patients[14-16]. However, there is still insufficient high-quality clinical evidence to support its effectiveness due to small sample sizes, unclear reporting and potential bias[17]. We hypothesized that Acupuncture plus conventional treatment would be an effective treatment for PD patients with poor sleep quality compared with sham acupuncture. Therefore, we design this study to investigate the efficacy of Acupuncture for treating PD with poor sleep quality. The trial not only compared the effectiveness of real acupuncture (RA) versus sham acupuncture (SA) but also provided a comprehensive evaluation of the relief of sleep quality, anxiety level, NMS, motor symptoms, and HRQoL in PD patients experiencing comorbid insomnia and motor symptoms.

2.3 Study Objective

To assess the safety and efficacy of real acupuncture versus sham acupuncture as adjunctive therapy for

PD patients with poor sleep quality.

2.4 Methodology

2.4.1 Trial Design

This was a single-center, randomized, controlled clinical trial with blinded participants, outcome assessment, and statistician in China. This study received approval from the Ethics committee of the First Affiliated Hospital of Guangzhou University of Chinese Medicine (Approval No. K [2021]104), and written informed consent was obtained from all participants. This trial was registered with Chinese Clinical Trial Registry (ChiCTR2200060655). This study followed the Consolidated Standards of Reporting Trials (CONSORT) reporting guideline.

2.4.2 Patients

2.4.2.1 Patients Recruitment, Screening

The participants will be recruited from the Parkinson clinic of the First Affiliated Hospital of Guangzhou University of Chinese Medicine. Interested participants can contact the researchers through the provided telephone numbers or WeChat code. An independent researcher will conduct face-to-face interviews with the participants to explain the study, and those who volunteer to participate will be required to sign consent forms (Appendix 1). After a baseline screening visit, participants who meet the inclusion criteria will be able to participate in the study.

2.4.2.2 Inclusion Criteria

(1) patients diagnosed with idiopathic Parkinson’s disease according to the MDS clinical diagnostic criteria for Parkinson's disease published in 2015[18].

(2) self-reported moderate or severe sleep problems or a Parkinson’s Disease Sleep Scale (PDSS) score between [0,100].

(3) aged 30 to 80 years old.

(4) stages 1 to 3 of Hoehn and Yahr(H&Y) scale.

(5) acceptance of acupuncture therapy.

(6)stable use of anti-Parkinson medication for 30 days.

(7) understanding of the protocol and signing the informed consent form.

2.4.2.3 Exclusion Criteria

(1) patients who did not meet any of the above criteria.

(2)patients unable to cooperate normally due to severe cognitive dysfunction, blindness, deafness, etc.

(3)comorbid with some other serious systemic diseases such as stroke, malignancy, renal failure, diabetes mellitus, atrial fibrillation, and so on.

(4)irregular use of sleep-assisted medication.

(5)received acupuncture treatment within the last 30 days.

(6) drug or alcohol abuse history.

(7) pregnancy or lactation.

2.4.3 Trial Flow

2.4.3.1 Screening Visit

Patients will be screened according to inclusion and exclusion criteria, eligible patients will be required to provide written informed consent and enter the baseline visit. Randomization will be performed before baseline assessment (2.4.4).

2.4.3.2 Baseline Visit

Researchers give participants sleep hygiene education, tell them keep the initial anti-parkinson medication and assess their demographic, clinical characteristics and observation scales.

2.4.3.3 Acupuncture treatment

Treatment will begin after baseline assessment. The two groups' participants will receive treatment for 4 weeks, three times per week (every Monday, Wednesday, Friday). Check for adverse events and concurrent drugs.

2.4.3.4 Aftertreatment Visit

After 12 times treatment, evaluators assessed and recorded scale outcomes, UPDRS scale, Hoehn-Yahr scale, PDSS scale, ESS scale, and PDQ39 scale. Adverse events are also assessed and recorded.

2.4.3.5 Follow-up and Endpoint

The follow-up visits will be arranged for 4 weeks after the end of treatment. Patients will receive a final assessment of scales , adverse events and concomitant medications at the week 8.

2.4.3.6 Patient Withdrawals

Patients may leave the study at their own discretion, and the investigator may determine if the patients withdraw from the trial due to violation of the trial protocol or the occurrence of a serious adverse event. Figure1 shows the flow diagram of the trial.

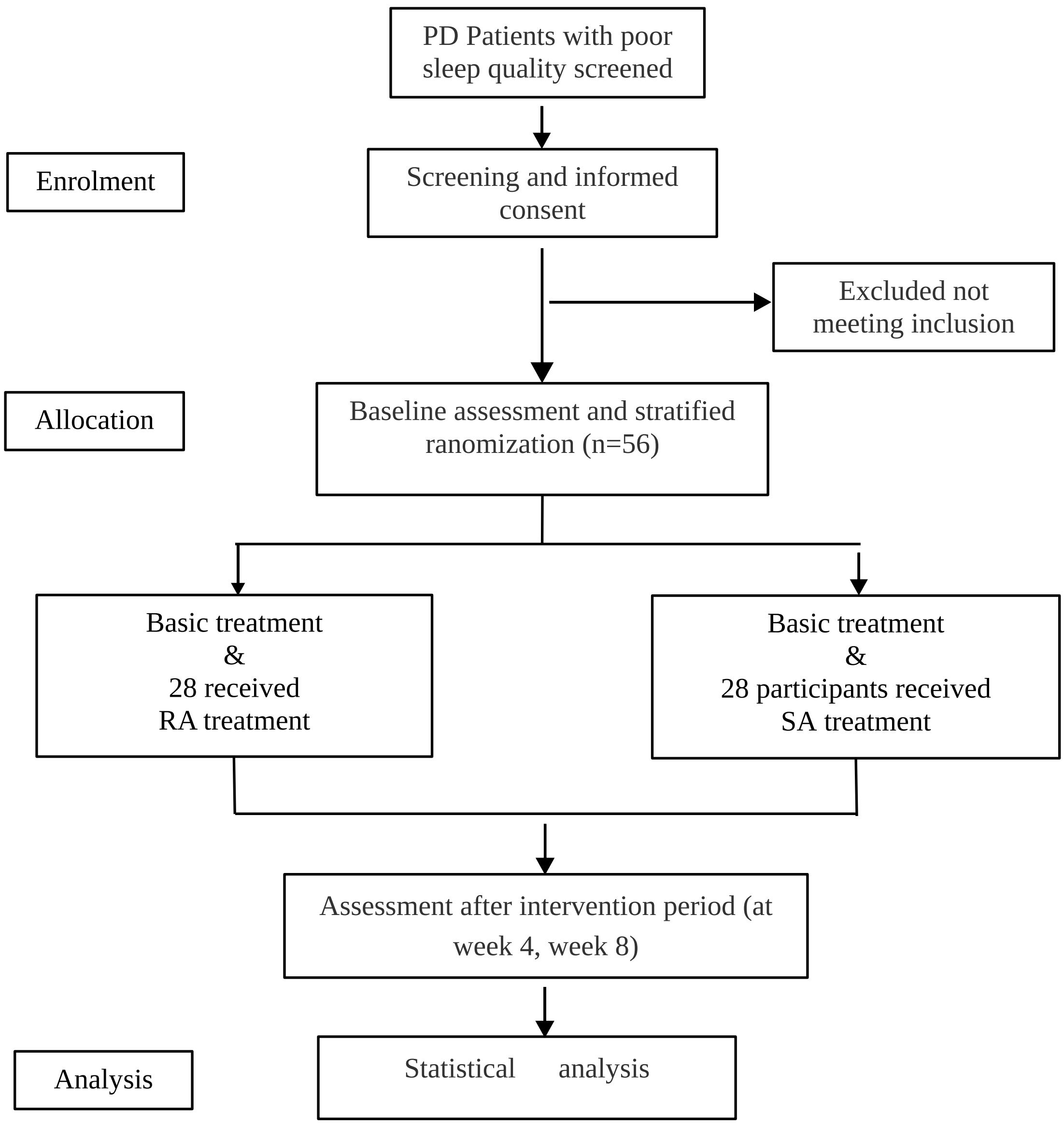

Figure 1 Flow chart

2.4.4 Randomization and Allocation Concealment

Patients were randomly assigned at random (1:1) to either the SA group or the RA group. A randomized sequence was generated using SPSS Statistics 26.0 (IBM SPSS Inc, Chicago, USA), the allocation sequence was hidden from the investigators, the allocation sequence was contained in sequentially numbered sealed opaque envelopes and managed by a third party (not the investigators). When a participant met the 8inclusion criteria, the clinical research coordinator contact with the third party to get his random number and group assignment. The acupuncturist will give the corresponding intervention to the subject.

2.4.5 Blinding

This clinical trial blinded participants, outcome assessment, and statisticians in order to prevent bias. Due to the nature of acupuncture and the necessity of this study, the acupuncturists were not blinded to the treatment allocation. And we design an equipment to assist in blinding participants. Acupuncturists were instructed not to disclose the patient's group assignment to patients and data collectors at any time.

2.4.6 Sample Size

The purpose of our study was to compare the improvement of sleep quality in PD patients between the SA groups and the RA group. Sample size was based on the pre-experiment PDSS score, with mean(SD) PDSS score s of 122.27 (15.35) for acupuncture and 100.4 (21.4) for sham acupuncture subjects. To achieve 90% power at a two-side significance of $p < 0.05$, a sample size of 44 subjects (22 per group) was calculated. Considering a 20% dropout the sample size was 56 patients calculated by PASS software (version 15.0.5).

3. Interventions

3.1 Basic treatment

- **Anti-Parkinson medication** Due to the complexity of the clinical presentation symptoms of PD and the principle of personalized treatment in PD clinical guidelines, PD treatment was strictly conducted in this trial. Madopa is the major medicine utilized in the treatment of PD patients. However, the application of these medication can affect patient's motor and non-motor symptoms, including sleep disorders. So we request if the subjects is taking dopaminergic medication regularly before enrollment, they can continue to take an effective dose medication but the dose cannot be modified at will. To solve this issue, the dosages of the different drugs will be converted to a total daily levodopa equivalent dose (LED) and recorded by data assessors.[17]

- **Sleep hygiene education** Based on the sleep symptoms of the patient, regular sleep hygiene education was provided to patients to help them develop correct perceptions and establish proper sleep habits. Patients enrolled in the trial had opportunity to discuss their specific sleep problems with researchers in a 30-60 minutes face-to-face evaluation. Researchers introduced sleep relaxation techniques and recommended them books related with sleep hygiene. After that, researchers are requested to contact participants every 2 weeks to follow and address subjects' sleep questions.

3.2 Real Acupuncture (RA groups)

- **Duration** The RA group received thirty-minute sessions of treatment three times each week for four weeks, for a total of 12 sessions.
- **Acupoints** The bilateral connection of Si Shenzhen, ShenTing(GV24), YinTang(GV29), HeGu(LI4), TaiChong(LR3), SanYinJiao(SP6), ShenMen(HT7), ZuSanLi(ST36), ShenMai(BL62), ZhaoHai(KI6). The acupoint names and locations adhered to the National Standard of the People's Republic of China: Nomenclature and location of meridian points (GB/T 12346–2021), established in 2021. All treatments were performed by senior acupuncturists (≥2 years of service), who used the same standardized equipment.
- **Procedure** Patients lay flat on the treatment bed and wore opaque eye masks. After confirming the acupuncture points, doctors firstly pressed the needle holder tightly to the skin of the acupoints and then performed acupuncture, inserting sterile needles into the corresponding 15°/90° needle entry portals on the needle holders. This procedure lasted 30 minutes and then doctors took out needles and needle holders and gently pressed needle holes with a sterilized dry cotton swab.
- **Equipments** Sterile acupuncture needles (Φ0.30mm×25mm or Φ0.30mm×40mm, Tianxie, Suzhou Medical Appliance Factory, China, depending on different patient's body), a new auxiliary acupuncture device for RA group (shown in figure2,figure 3)
- **Technique** Different acupoints require different approaches for inserting needles because of different muscle distribution: shallower parts require flat stabbing, with the needle tip and skin at an angle of 15 °, richer parts require straight stabbing into the tissue, with the needle tip and skin at an angle of 90 °. All four Sishenzhen points and Shenting point were oriented toward the top of the head, the orientation of Yintang were downward. All these acupoints on the head was entered flatly for 15~20 mm. The points of shenmen should be stimulated straightly for 12-20 mm but the superficial veins should be avoided. The needle was stimulated straightly for 20-30 mm in Sanyinjiao and Zusanli point, and slightly downward diagonal stabbing for 10-15mm in Shenmai and Zhaohai. Deqi (a sensation of aching, soreness, swelling, heaviness, or numbness) was then obtained by manipulation.

3.3 Sham Acupuncture (SA group)

The SA group applied the same intervention duration, acupoints and procedure with the RA group, but different acupuncture equipment.

- **Equipment** Blunt sterile acupuncture needles (Φ0.30mm×25mm or Φ0.30mm×40mm, Tianxie, Suzhou Medical Appliance Factory, China, depending on different patient's body), a new auxiliary acupuncture device for SA group. (shown in figure2,figure 3)
- **Technique** the orientation and angle were the same as the RA group. Patients may feel a pinprick sensation but the blunt needle didn't penetrate the skin into the subcutaneous tissue.

3.4 the Auxiliary Acupuncture Device

Our team designed an auxiliary device for sham acupuncture research, allowing for blinding patients effectively. This device for real and sham acupuncture share the same appearance, both are composed of a hemispherical base and a telescopic tube. The insertion angle of needles can be adjusted from 15° to 165° by rotating the telescopic tube in the notch of the base. The bottom of the base is attached with hydrogel, which has good fixity. The auxiliary device is applicable to multiple parts of the human body and can effectively reduce the risk of unblinding. The acupuncturists adopts a tube-needle approach to insert needle, tapping the inner tube's tip quickly with the finger before inserting the needle downward. the tip of the needle pierced the hemispherical silicone needle cushion inside the SA group, and the needle tip pierced the human skin inside the RA group.

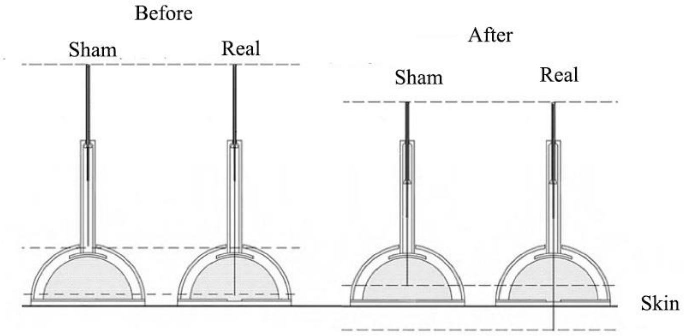

Figure2 the Schematic Diagram of Auxiliary Acupuncture Device

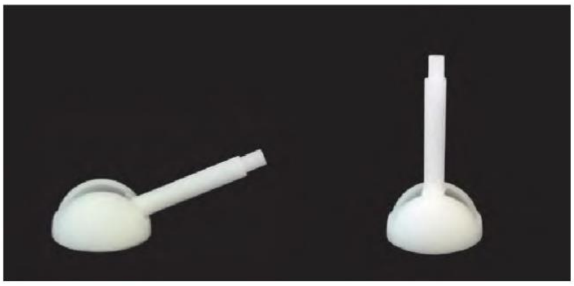

Figure 3 the Auxiliary Acupuncture Device

4. Outcome Measurements

4.1 Primary Outcomes

Our primary outcome was the was change from baseline on the Parkinson’s Disease Sleep Scale (PDSS) assessed 3 times at week 0,week 4 and week 8, before, after treatment and follow-up. The PDSS is a 15-item self-report scale that gauge the impact of poor sleep quality on various functions, evaluating 8 aspects of nocturnal sleep in PDSS. These aspects include the overall quality of a night's sleep,

insomnia, nocturnal restless, nocturnal psychosis , nocturia, nocturnal motor symptoms, sleep refreshment, and daytime dozing. The assessed period is “the past 1 week”, with patients marking their response to each item on a visual analogical scale ranging from “always” (0) to “never”(10). The total PDSS score ranges from 0 to 150. And scale score increases as their quality of sleep improves.

4.2 Secondary Outcomes

The secondary outcomes included the completion rate, adverse events, and assessment of participants with the following protocol: (1) the severity of motor symptoms were quantified by modified Hoehn-Yahr Scale and the Unified Parkinson's Disease Rating Scale(UPDRS, UPDRS-III),daily levodopa equivalent dose(LED).(2) the overall severity of motor symptoms was assessed by Non-Motor Symptoms Scale (NMSS). Specifically, excessive daytime sleepiness was assessed by Epworth Sleepiness Scale (ESS), anxiety levels were assessed by the Hamilton Anxiety Rating Scale (HAMA). (3) Quality of life (QOL) was assessed by the 39-item Parkinson Disease Questionnaire (PDQ-39). Key secondary outcomes were also assessed 3 times at week 0, 4 and 8.

The research process and data collection are described in detail in Table 1.

Table 1 Schedule of enrolment, interventions, and assessments

Study period			Intervention period		Follow-up period
Time point			week 0	Week 4	Week8
Eligibility screening			×		
Informed consent			×		
Randomization			×		
Primary outcome		PDSS	×	×	×
Secondary outcomes	Assessment of motor symptoms	UPDRS	×	×	×
		UPDRS-Ⅲ	×	×	×
		LED	×	×	×
	Assessment of non-motor symptoms	NMSS	×	×	×
		ESS	×	×	×
		HAMA	×	×	×
	Quality of life	PDQ-39	×	×	×
			×	×	×
	AEs			×	×
Compliance evaluation				×	×

Abbreviations: PD, Parkinson’s disease; PDSS, Parkinson’s Disease Sleep Scale; ESS, Epworth Sleepiness Scale; UPDRS, the Unified Parkinson's Disease Rating Scale; UPDRS-III, the section 3 of the Unified Parkinson's Disease Rating Scale; NMSS, the Non-Motor Symptoms Scale; HAMA, the Hamilton Anxiety Rating Scale; PDQ-39, 39-item Parkinson Disease Questionnaire.

5. Safety Assessment

AE is defined as feeling unwell during the trial related to treatment of PD or acupuncture treatment. Throughout all the treatment visits and the follow-up visits, we monitored the treatment-induced adverse events, recording adverse events such as needle sickness, needle breakage, hematoma, infection, etc. All patients will directly report any adverse event (mild, moderate, or severe) to the acupuncturists. Common AEs related to acupuncture include fainting, local ecchymoses, continuous pain, acupoint location infection and dizziness, while AEs related to PD treatment include severe

tremor, nausea, vomiting, hypotension. A serious adverse event was defined as any adverse event posing a threat to a patient’s life or functioning. The severity of adverse events (mild, moderate, or severe) was assessed by the investigators. We ask patients in both groups to call acupuncturists to report any discomfort during the trial, and the acupuncturists need to ask in detail and determine their relevance to treatment. Acupuncturists should evaluate the patient's condition to decide whether the treatment can be continued if they happen. The results of AEs will be described as the number and proportion (%) of AEs.

6. Ethical Principle

This study protocol has been approved by ethics committees of the First Affiliated Hospital of Guangzhou University of Chinese Medicine (Approval No. K [2021]104). This study conforms to the Declaration of Helsinki principles. Patient enrollment won't start until the Institution Review Board (IRB) approves the trial protocol, but everything should happen following registration.

7. Statistical Analysis

Shapiro-Wilk normality analysis was applied at baseline, with results conveyed as mean (SD) and the t-test for normal distributions, or as Median (%) and the Mann-Whitney U test for non-normal distributions. Group differences in trial outcomes were assessed using a linear model for continuous variables. Key estimates and standard errors of therapeutic assessment between groups were obtained through the PROC MIXED data procedure in SAS statistical software, version 9.4. Specifically, a mixed-effects model was employed to evaluate the primary outcome of PDSS, as well as secondary outcomes including ESS, UPDRS, UPDRS-III, NMSS, HAMA, and PDQ-39, considering the interaction effects between time and group.

Missing data were addressed using multiple imputations with the Markov chain Monte Carlo method. Efficacy was assessed in full analysis set, which included all randomized patients who received at least one week of acupuncture. Continuous variables were presented as least-squares means with 95% confidence intervals(CI). It is essential to note that the 95% CI were not adjusted for multiple comparisons and should not be utilized to infer definitive treatment effects. A two-sided P value of less than 0.05 was considered to indicate statistical significance for the primary outcome. All analyses were performed using SAS software (version 9.4; SAS Institute Inc. Cary, NC).

8.Some revisions

We have made certain changes to the initial trial protocol to properly conduct this trial and reflect the effectiveness of acupuncture from a wider range of perspectives. Compared with the original plan, we

have reduced the frequency of intervention from 4 times a week to 3 times a week, to avoid the traffic and time burden caused by commuting to and from the hospital. We used PDQ-39 instead of SF-36 to evaluate the patient health-related quality of life, because PDQ-39 is more targeted in Parkinson patients. In order to provide comprehensive results, we added some scales as secondary outcomes: UPDRS to evaluate the severity of motor symptoms, HAMA to evaluate the anxiety level, NMSS to evaluate the overall severity of non-motor symptoms. The Rapid-eye-movement Sleep Behavior Disorder Screening Questionnaire (RBDSQ) was eliminated because there were insufficient data to determine its statistical efficacy. We ultimately gave up on serological testing because of issues with reagent transportation and storage as well as patient compliance.

9. Funding

This work was supported by the National Natural Science Foundation of China (Grant Number. 82174486).

10. References

1.Kalia LV, Lang AE. Parkinson's disease. Lancet. 2015;386(9996):896-912.
2.Heimrich KG, Schönenberg A, Santos-García D, Mir P, Coppadis Study G, Prell T. The Impact of Nonmotor Symptoms on Health-Related Quality of Life in Parkinson's Disease: A Network Analysis Approach. J Clin Med. 2023;12(7).
3.Martinez-Martin P. Nonmotor symptoms and health-related quality of life in early Parkinson's disease. Mov Disord. 2014;29(2):166-168.
4.Marinus J, Zhu K, Marras C, Aarsland D, van Hilten JJ. Risk factors for non-motor symptoms in Parkinson's disease. Lancet Neurol. 2018;17(6):559-568.
5.O'Dowd S, Galna B, Morris R, et al. Poor Sleep Quality and Progression of Gait Impairment in an Incident Parkinson's Disease Cohort. J Parkinsons Dis. 2017;7(3):465-470.
6.de Almeida FO, Ugrinowitsch C, Brito LC, et al. Poor sleep quality is associated with cognitive, mobility, and anxiety disability that underlie freezing of gait in Parkinson's disease. Gait Posture. 2021;85:157-163.
7.Mao CJ, Yang YP, Chen JP, et al. Poor nighttime sleep is positively associated with dyskinesia in Parkinson's disease patients. Parkinsonism Relat Disord. 2018;48:68-73.
8.Gómez-Esteban JC, Zarranz JJ, Lezcano E, et al. Sleep complaints and their relation with drug treatment in patients suffering from Parkinson's disease. Mov Disord. 2006;21(7):983-988.
9.Bailey GA, Hubbard EK, Fasano A, et al. Sleep disturbance in movement disorders: insights, treatments and challenges. J Neurol Neurosurg Psychiatry. 2021;92(7):723-736.
10.Shafazand S, Wallace DM, Arheart KL, et al. Insomnia, Sleep Quality, and Quality of Life in Mild

to Moderate Parkinson's Disease. Ann Am Thorac Soc. 2017;14(3):412-419.
11.Tsuru A, Matsui K, Kimura A, et al. Sleep disturbance and health-related quality of life in Parkinson's disease: A clear correlation between health-related quality of life and subjective sleep quality. Parkinsonism Relat Disord. 2022;98:86-91.
12.Liu CF, Wang T, Zhan SQ, et al. Management Recommendations on Sleep Disturbance of Patients with Parkinson's Disease. Chin Med J (Engl). 2018;131(24):2976-2985.
13.Videnovic A. Management of sleep disorders in Parkinson's disease and multiple system atrophy. Mov Disord. 2017;32(5):659-668.
14.Cheng FK. The use of acupuncture in patients with Parkinson's disease. Geriatr Nurs. 2017;38(4):302-314.
15.Kluger BM, Rakowski D, Christian M, et al. Randomized, Controlled Trial of Acupuncture for Fatigue in Parkinson's Disease. Mov Disord. 2016;31(7):1027-1032.
16.Zeng BY, Zhao K. Effect of Acupuncture on the Motor and Nonmotor Symptoms in Parkinson's Disease--A Review of Clinical Studies. CNS Neurosci Ther. 2016;22(5):333-341.
17.Hsu WT, Hsu CM, Hung SC, Hung SY. Acupuncture Improves Sleep Disorders and Depression among Patients with Parkinson's Disease: A Meta-Analysis. Healthcare (Basel). 2023;11(14).
18.Postuma RB, Berg D, Stern M, et al. MDS clinical diagnostic criteria for Parkinson's disease. Mov Disord. 2015;30(12):1591-1601.

Appendix 1

Informed Consent Form-Information Page

Dear participant:

Your doctor has diagnosed you with Parkinson's disease and you are disturbed by sleep problems.

You are invited to participate in a study titled “Acupuncture for Parkinson's disease patients with poor sleep quality: A randomized, controlled trial”.

Please read the following information as carefully as possible before you decide whether to participate in this study. It can help you understand why this study is being done, the procedures and duration of the study, the benefits, the risks you may face, and the discomfort you may experience if you participate in the study. You can discuss it with your relatives, friends, or ask your doctor for an explanation to help you make a decision.

Study background and objectives

Parkinson's disease (PD) is a kind of progressive neurodegenerative disease commonly in people over 50 years old. With the increase in the percentage of ageing people in the global population, the incidence of PD is also increasing. The prevalence was 17/100 000 in people over 65 years old in China. PD patients, experiencing poor sleep quality and quantity, typically come to hospitals with sleep complaints and more severe motor conditions and negative health-related quality of life. If PD patients can be diagnosed and treated in a timely manner at an early stage, they can often get better treatment effects, meet the basic work requirements and obtain better quality of life.

Acupuncture, as a traditional Chinese treatment method, has gained global popularity in recent decades. It can play an important role in reducing adverse reactions to medicine and relieving non-motor symptoms. Acupuncture is often used to treat sleep disorders and PD. However, there is still insufficient high-quality clinical evidence to support its effectiveness due to small sample sizes, unclear reporting and potential bias. Therefore, Therefore, we conducted a randomized clinical trial to evaluate the efficacy and safety of acupuncture for poor sleep quality among PD patients.

This study will be conducted at the Parkinson clinic of **the First Clinical College of Guangzhou University Of Chinese Medicine.**

If you agree to take part in the study, you will need to do the following:

Before you are enrolled in the study, you will undergo some examinations to determine whether you can participate in the study. The doctor will ask about your medical history. A general physical examination will be conducted as well.

If you meet the inclusion criteria, you will be assigned to the acupuncture group or the control group for treatment according to the randomization. Patients in the study have a 50% chance of being assigned to either group. If you enroll this clinical experiment, please maintain your initial dose of anti-Parkinson medication.

Interventions in two groups

Real acupuncture group: maintain initial dose of anti-Parkinson medication for the treatment of PD, acupuncture treatment will be performed: three times per week from week 1 to 4. Patients need be assessed at week0, week 4, week 8.

Sham acupuncture group: maintain initial dose of anti-Parkinson medication for the treatment of PD, sham acupuncture treatment will be performed: three times per week from week 1 to 4. Patients need be assessed at week0, week 4, week 8.

Possible benefits of participating in the study

You and society probably benefit from this research. Your condition and quality of life may improve. This study may have a good guiding effect on clinical treatment. However, it is not excluded that this trial may not improve your condition.

Possible discomfort and inconvenience of participating in the study

If you experience any discomfort during the study period, changes in your condition, or any unexpected circumstances, whether it related to treatment or not, you should inform your doctor immediately. The doctor will make a judgment and initiate medical treatment as needed. During acupuncture clinical trials, some adverse events may occur, such as fainting, local ecchymoses, continuous pain, acupoint location infection and dizziness and so on. When fainting occurs, the needle will be removed immediately. The participant will be asked to lie flat with his/her head slightly lower than their body. The participant will be given warm boiled water or sugar water. In general, the participants will recover after lying for a while.

During the study period, you need to come to our clinic on time for treatment and follow-up, and

do some physical tests. These may cause trouble or inconvenience to you.

Personal information confidential

Your medical records (CRF, physical and chemical examination reports, etc.) will be kept in the hospital. Researchers, sponsor representatives, and ethics committees will be allowed access to your medical records. Any public report on this study will not disclose your personal identity. We will make every effort to protect the privacy of your personal medical data within the scope permitted by law.

Voluntary principle

You are voluntarily choose to participate in the study or drop out of the study. Participation in the study depends entirely on your willingness. You may refuse to participate in the study or withdraw from the study at any time during the study without affecting your relationship with your doctor, loss of your medical treatment or other benefits.

Your doctor or researcher may discontinue your participation in this study at any time for your best interest.

If you withdraw from the study for any reason, you may be asked about the treatment you are taking in the study. You may also be asked to perform physical examinations if your doctor thinks it is needed.

If you do not participate in this study, or withdraw from the study, there are many other alternatives, such as exercise therapy, surgery, and so on. You do not have to choose to participate in this study in order to treat your illness.

If you choose to participate in this study, we hope that you will be able to complete the entire study process.

Informed Consent Form-Signature Page

Clinical study project name: Acupuncture for Parkinson's disease patients with poor sleep quality: A randomized, controlled trial

Research unit: the Parkinson clinic of the First Clinical College of Guangzhou University Of Chinese Medicine, Guangzhou University of Chinese Medicine

Approval number : K [2021]104

Voluntary Subject Statement:

I have read the above introduction to this study and had the opportunity to discuss and ask questions about this study with my doctor. All my questions were satisfactorily answered.

I am aware of the risks and benefits of participating in this study. I understand that participation in the study is voluntary. I confirm that there is sufficient time to consider this and I understand that:

I can always ask my doctor for more information.

I can withdraw from the study at any time without being discriminated against or penalised and my medical rights and treatment will not be affected.

I am also aware that if I drop out of the study, especially I drop out of the study due to treatment reasons, it will be very beneficial to me and the study if I tell the doctor about the change in my condition and complete the required physical examination.

If I need to take any other treatment due to the change in my condition, I will consult my doctor in advance or inform him/her afterwards.

I grant access to my research materials to the ethics committee or the sponsor's representative.

I will receive a signed and dated copy of the informed consent. In the end, I agree to participate in the study and will try to follow my doctor's advice.

Subject's signature: Date:

Subject contact number:

Signature of subject's guardian: Date:

Guardian's contact number:

Subject's guardian is required to sign the informed consent if necessary.

Doctor's declaration

I have fully explained this study in detail to the above participant, including his/her rights and possible benefits and risks, and I have answered all his/her questions. To the best of my knowledge, the participant has been informed adequately and has consented to the trial.

Doctor’s signature: Date:

Doctor contact number:

In the event of inconsistency or discrepancy between the Chinese version and the English version, the Chinese language version shall prevail.
