# Supplementary material for: Acupuncture and Sleep Quality Among Patients With Parkinson Disease: A Randomized Clinical Trial
Source: JAMA Netw Open. 2024 Jun 26;7(6):e2417862. doi: 10.1001/jamanetworkopen.2024.17862 (PMC11208974; doi:10.1001/jamanetworkopen.2024.17862)
Supplement: Supplement 2. — eFigure 1. Schematic Diagram of the Auxiliary Acupuncture Device eFigure 2. Auxiliary Acupuncture Device eFigure 3. Acupoint Locations eFigure 4. Therapeutic Effects of Acupuncture eTable 1. Procedure Diagram eTable 2. Blinding Assessment eTable 3. Primary and Secondary Outcomes eTable 4. Acupuncture-Related Adverse Events [file jamanetwopen-e2417862-s002.pdf]

Supplementary Online Content

Yan M, Fan J, Liu X, et al. Efficacy of acupuncture for patients with Parkinson disease who have poor sleep quality: a randomized clinical trial. *JAMA Netw Open*. 2024;7(6):e2417862. doi:10.1001/jamanetworkopen.2024.17862

eFigure 1. Schematic Diagram of the Auxiliary Acupuncture Device

eFigure 2. Auxiliary Acupuncture Device

eFigure 3. Acupoint Locations

eFigure 4. Therapeutic Effects of Acupuncture

eTable 1. Procedure Diagram

eTable 2. Blinding Assessment

eTable 3. Primary and Secondary Outcomes

eTable 4. Acupuncture-Related Adverse Events

This supplementary material has been provided by the authors to give readers additional information about their work.

© 2024 Yan M et al. *JAMA Network Open.*

eFigure 1. Schematic Diagram of the Auxiliary Acupuncture Device

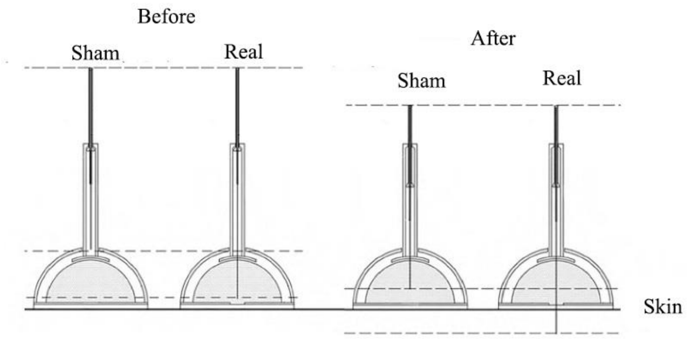

eFigure 2. Auxiliary Acupuncture Device

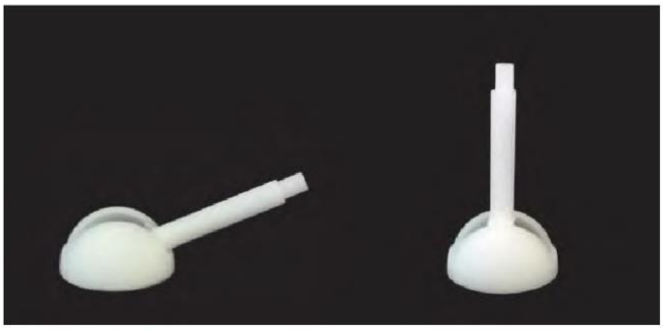

© 2024 Yan M et al. *JAMA Network Open.*

eFigure 3. Acupoint Locations

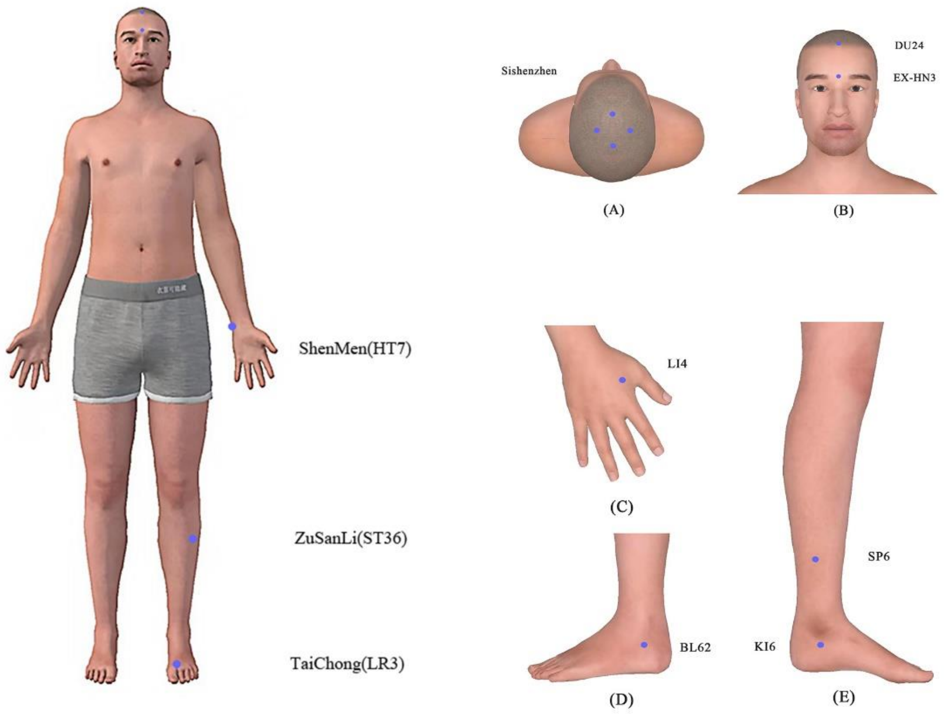

eFigure 4. Therapeutic Effects of Acupuncture

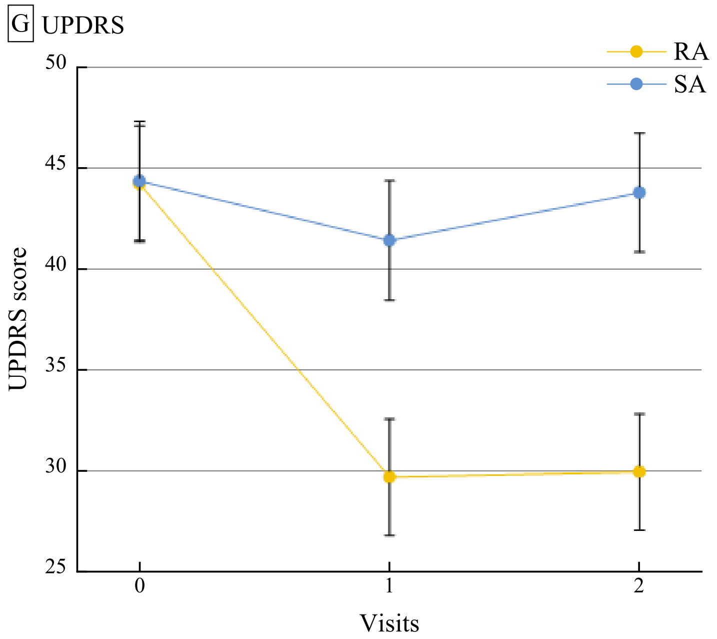

G, improvement in UPDRS score

© 2024 Yan M et al. *JAMA Network Open.*

eTable 1. Procedure Diagram

Study period			Intervention period		Follow-up period
Time point			week 0	Week 4	Week8
Eligibility screening			×		
Informed consent			×		
Randomization			×		
Primary outcome		PDSS	×	×	×
Secondary outcomes	Assessment of motor symptoms	UPDRS	×	×	×
		UPDRS-Ⅲ	×	×	×
		LED	×	×	×
	Assessment of non-motor symptoms	NMSS	×	×	×
		ESS	×	×	×
		HAMA	×	×	×
	Quality of life	PDQ-39	×	×	×
			×	×	×
	AEs			×	×
Compliance evaluation				×	×

eTable 2. Blinding Assessment

Group	Acupuncture (n)	Sham acupuncture (n)	κ
Real acupuncture group(n=40)	23	17	0.154
Sham acupuncture group(n=38)	16	22	

Note: Cohen's kappa of the Auxiliary Acupuncture Device

© 2024 Yan M et al. *JAMA Network Open.*

eTable 3. Primary and Secondary Outcomes

Domin			Significance
Primary outcome		PDSS	nocturnal sleep quality, scores range from 0 to 150, with higher scores indicating better sleep quality and less sleep disorders.
Secondary outcomes	Assessment of motor symptoms	UPDRS	Overall assessment, scores range from 0 to192, with higher scores indicating more severe overall symptoms of Parkinson's disease
		UPDRS-III	Motor status, cores range from 0 to 56 , with higher scores indicating more severe motor symptoms of Parkinson's disease
		LED	Levodopa equivalent dose conversion
	Assessment of non-motor symptoms	NMSS	Overall NMS assessment, scores range from 0 to120, with higher scores indicating more severe non-motor symptoms of Parkinson's disease
		ESS	Daytime sleepiness level, Scores range from 0 to 24, with higher scores indicating more serious sleepiness.
		HAMA	Anxiety state, a Scores range from 0 to 56, with higher scores indicating more serious anxiety
	Quality of life	PDQ-39	Scores range from 0 to156, with higher scores indicating worse quality of life

eTable 4. Acupuncture-Related Adverse Events

**Adverse Event**	**RA (n=40)**		**SA(n=38)**	
	**Participants,*n(%)***	**Events,*n***	**Participants,*n(%)***	**Events,*n***
Needle left in participant	0	0	0	0
Nausea	1(2.5)	1	0	0
Fainted	0(0)	0	0	0
Severe sharp pain	4(10)	6	1(2.6)	0
Sharp pain lasting >10min	1(2.5)	3	0	0
Hematoma	3(7.5)	5	0	0
Bleeding/numbness/infection	5(12.5)	5	0	0
Severe tremor during treatment	7(17.5)	7	9(23.7)	9
allergy	2(5)	2	1(2.6)	0
Other discomforts after acupuncture	3(7.5)	3	1(2.6)	1

© 2024 Yan M et al. *JAMA Network Open.*
