# Supplementary material for: Acupuncture and Sleep Quality Among Patients With Parkinson Disease: A Randomized Clinical Trial
Source: JAMA Netw Open. 2024 Jun 26;7(6):e2417862. doi: 10.1001/jamanetworkopen.2024.17862 (PMC11208974; doi:10.1001/jamanetworkopen.2024.17862)
Supplement: Supplement 3. — Data Sharing Statement [file jamanetwopen-e2417862-s003.pdf]

Data Sharing Statement

Yan. Acupuncture and Sleep Quality Among Patients With Parkinson Disease. *JAMA Netw Open*. Published June 26, 2024. doi:10.1001/jamanetworkopen.2024.17862

Data
Data available: No

Additional Information
Explanation for why data not available: If there is any unavailable data in the article, please get in touch with the corresponding author; information will be provided upon reasonable request.
